# Design of Embedded System for Multivariate Classification of Finger and Thumb Movements Using EEG Signals for Control of Upper Limb Prosthesis

**DOI:** 10.1155/2018/2695106

**Published:** 2018-05-20

**Authors:** Nasir Rashid, Javaid Iqbal, Amna Javed, Mohsin I. Tiwana, Umar Shahbaz Khan

**Affiliations:** Department of Mechatronics Engineering, National University of Sciences & Technology, H-12, Islamabad, Pakistan

## Abstract

Brain Computer Interface (BCI) determines the intent of the user from a variety of electrophysiological signals. These signals, Slow Cortical Potentials, are recorded from scalp, and cortical neuronal activity is recorded by implanted electrodes. This paper is focused on design of an embedded system that is used to control the finger movements of an upper limb prosthesis using Electroencephalogram (EEG) signals. This is a follow-up of our previous research which explored the best method to classify three movements of fingers (thumb movement, index finger movement, and first movement). Two-stage logistic regression classifier exhibited the highest classification accuracy while Power Spectral Density (PSD) was used as a feature of the filtered signal. The EEG signal data set was recorded using a 14-channel electrode headset (a noninvasive BCI system) from right-handed, neurologically intact volunteers. Mu (commonly known as alpha waves) and Beta Rhythms (8–30 Hz) containing most of the movement data were retained through filtering using “Arduino Uno” microcontroller followed by 2-stage logistic regression to obtain a mean classification accuracy of 70%.

## 1. Introduction

A Brain Computer Interface (BCI) provides a communication system to control external device(s) in which messages or commands are sent to external world through brain signals. These signals do not pass through the brain's normal output pathways of nerves and muscles. Rather, BCI provides an alternate method to its user to interact with the world. For example, Electroencephalogram (EEG) based BCI messages are encoded in EEG activity of brain. For people with amputation or severe neuromuscular disability, who may lack normal output channels, BCIs prove to be useful for controlling external devices [1]. The world of BCI is growing day by day, with applications ranging from control of upper/lower limb prosthesis and wheel chairs to control of multimedia applications and smart phones for people suffering from stroke [2, 3].  Table 1 shows some researches in which upper limb prosthesis or cursor is controlled using motor imagery. Movements of a prosthesis are commonly controlled through manipulating the motion of rotary actuator (electric motor) in a BCI system.

BCI system consists of input signals (electrophysiological activity recorded from scalp of user), a signal processor (filtering the signal for desired frequency and extracting features for best representation of user intent), a translating algorithm or classifier (that anticipates the human intent from the selected feature), and finally a control algorithm that controls the device attached to the system [1].

Mental activity, such as imagination of movement and movement itself or decision making, results in excitation of Neural Networks which cause changes in electrical potentials that can be recorded by sensors [2]. This electrical potential is recorded using invasive (placement of sensor under the scalp through surgery) or noninvasive (placement of sensors on the scalp) sensors. The invasive method provides a higher signal to noise ratio; however, it is cost-wise expensive and involves risk due to surgery. There is a variety of changes in electrical potentials that can be extracted from real time recorded EEG signals [10] which can be either evoked potentials or induced potentials. This includes Event Related Potentials (ERP), P300 Evoked Potentials [11], Slow Cortical Potentials, Visual Evoked Potentials, and Mu and Beta Rhythms over the sensorimotor cortex [12].

In this research, noninvasive electrode equipment is used for recording EEG signals form scalp. The EEG signal data set was recorded using a 14-channel electrode headset (Emotiv headset) from right-handed, neurologically intact volunteers. This research is a follow-up of our previous research in which Mu and Beta Rhythms (8–30 Hz) were used. Power Spectral Density (PSD) was used for analysis of filtered data followed by logistic regression for classification of finger movements [13]. PSD describes the distribution of power of signal over its frequency. The band power of Power Spectral Density [14] of finger movements of one hand occurring over the motor cortex is used as a feature to classify them. The Mu and Beta Rhythms that occur over the motor cortex provide us with information related to the movement [15, 16].

A variety of classification techniques are used in BCI systems such as Neural Networks (NN), Support Vector Machines (SVM), Discriminant Analysis, and Bayesian Classifiers. As an extension of our previous research logistic regression is used as a classifier, and output of the classifier is used for generating command signals to control upper limb prosthesis [17–19].

Our previous research [13] compared different classifiers, namely, Multilayer Perceptron, Linear Discriminant Analysis (LDA), Quadratic Discriminant Analysis (QDA), and logistic regression to achieve highest classification accuracy [20, 21]. Two-stage logistic regression gave the highest classification accuracy of 74% for four finger movements (thumb, index finger, index and middle finger combined, and fist). Weka 3.6.9 (data mining software with collection of machine learning algorithms) and Matlab were used to process the signals in earlier research.

In the current research our emphasis is on the use of embedded system to process EEG data for generating command signals for upper limb prosthesis. Arduino Uno is used as the embedded system to filter signals (between 8 and 30 Hz), extract features (PSD), and differentiate between three finger movements. In this research, we are using three targeted finger movements (thumb, index finger, and fist) instead of four (as were in our previous research). The reason to restrict ourselves to three movements only is that embedded system is not able to distinguish between the index finger movement and index-middle finger combined movement.

## 2. Materials and Methods

### 2.1. Section I: Experimental Protocol and Data Acquisition

**Figure 1:**
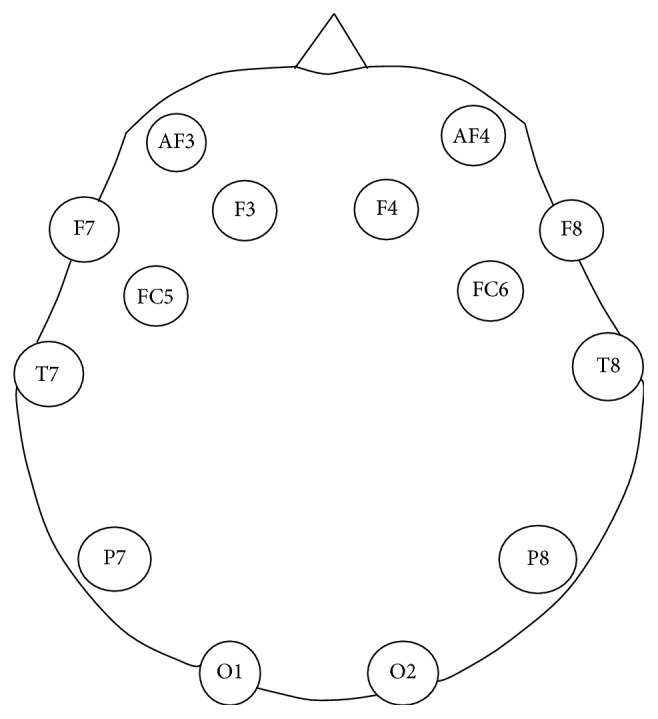
Electrode placement [13].

**Figure 2:**
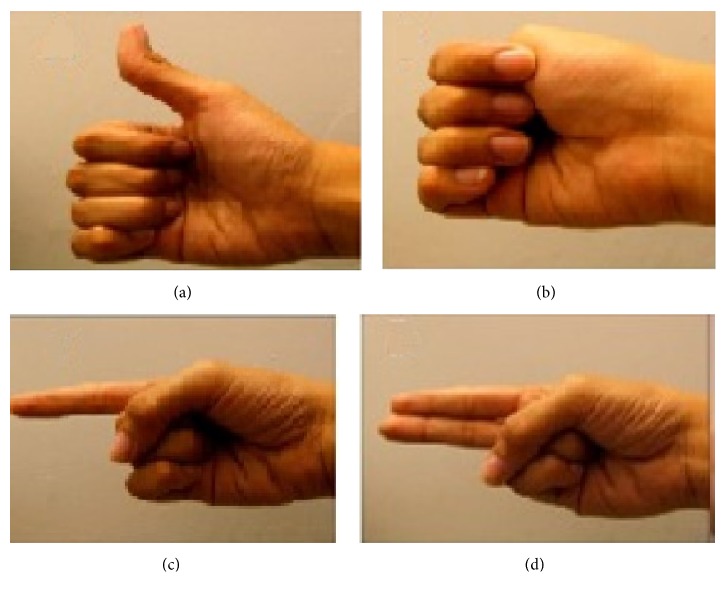
Finger movements that were recorded. (a) Thumb movement. (b) Fist movement. (c) Index finger movement. (d) Two-finger (index and middle) combined movement [13].

The data was acquired from four subjects (one female and 3 male) who volunteered to undergo data recording protocol. One of the male subjects (described as category I in Results) has a habit of high involuntary eye blinking frequency. The other three subjects are described as category II in Results. The age of subjects is between 22 and 45 years. The process of data acquisition from the subjects is approved by the departmental ethical review board. The volunteers are healthy with no known neurological disabilities. The data was acquired using Emotiv headset at 128 Hz sampling frequency. Emotiv has 14 noninvasive electrodes placed according to the international 10-20 system shown in Figure 1 [13]. The data was acquired for four movements, i.e., thumb, fist, index finger, and index-middle finger combined movements. The movements are shown in Figure 2 [13]. Three out of these acquired movements (thumb, index finger, and fist movement) data were used for this research.

**Figure 3:**
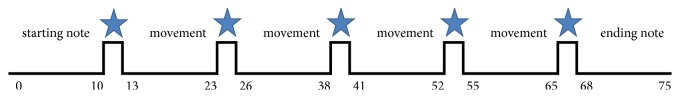
Data acquisition protocol.

During data acquisition, the subjects were comfortably sitting in a chair and were asked to perform the movements shown on the computer screen. For each subject the acquired data of one trial contained 10 seconds of data for each movement, which makes 1280 samples per movement. The total samples in one recorded trial are 1280 × 4 = 5120. There were a total of 60 recorded trials for each subject, out of which 13 trials for each subject were rejected on the basis of visual inspection. Rest of the 47 data trials for each subject were used. For this research samples of only three movements (thumb, index finger, and fist) were used instead of four movements. Thus, for this research 47 × 1280 × 3 = 180480 samples have been used for one subject. The data acquisition protocol is shown in Figure 3.

### 2.2. Section II: Embedded System

The aim of this research was to design an embedded system that can be used to classify and control upper limb prosthesis finger movements using acquired EEG signals. “Arduino Uno” is the embedded system used to fulfill the aim of this research. The attributes of embedded system (Arduino Uno) are given in Table 2.

**Figure 4:**
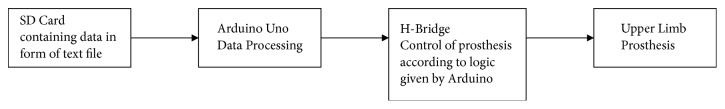
Stages of system from data input to device control.

The data was given as input to Arduino Uno, which was programmed to process the input (filtering and classification). Basing upon the result of classification, generate a signal that controls motors connected to upper limb prosthesis fingers. Stages of the system from data input to device control are shown in Figure 4.

Data processing steps included digital filtering with a high pass and low pass filter to retain 8–30 Hz of frequencies. Filtration was followed by feature extraction (calculation of band power from PSD of the remaining frequencies) from the data. The feature vector was then given as input to a logistic regression classifier network for classification of three finger movements. Based on the classification, a command signal is generated and sent to a motor drive circuitry (H-Bridge in this case) to actuate the respective motor to start the finger movement of upper limb prosthesis.

This research was carried out using data already acquired from the subjects. Data set of each trial consisted of 10 seconds of data of 14 channels at the sampling rate of 128 Hz. From each of the 47 trials, 250 ms of data was extracted and converted to text files. The data was saved offline on an SD card in text file and given as input to Arduino Uno. The SD card was interfaced with the controller using Serial Peripheral Interface (SPI). SPI operates in full duplex mode with a Master Out Slave In Pin, Master In Slave Out Pin, Serial Clock Pin, and Chip Select Pin. The Arduino acted as Master, while the SD card acted as Slave. The Arduino first enabled the SD card through Chip Select. The clock was set at a baud rate of 9600. The Pin configuration of connection between SD card and Arduino Uno is shown in Table 3.

As discussed earlier in this section, 250 ms of data was extracted from each trial, converted, and saved in text file. During processing, first 250 ms of data is read by the embedded system, processed, and classified. Then, next 250 ms of data of next trial is read and processed and the loop continues for classification until the data reaches its end.

### 2.3. Section III: Filtration Techniques

Filtering the data to extract Mu and Beta band of frequencies (8–30 Hz) is carried out as this band contains maximum information related to finger movements. To execute this through embedded system, 250 ms of EEG data was digitally filtered using a Butterworth filter between 8 and 30 Hz of order 2. The reason for using Butterworth filter was its flat response with zero ripples. The coefficients of the Butterworth filter were taken from Matlab “butter” command and are shown in Tables 4 and 5.

Filtration was done using these coefficients in the filter difference equation defined by [21]

(1) a1∗yn=b1∗xn+b2∗xn−1+b3∗xn−2−a2∗yn−1−a3∗yn−2,

where **y** is the output, **x** is the input, and **n** is the *n*th element of the output.

Before passing the data through filter, it was padded. Later, after the forward and reverse filtering, the data was truncated back to its original number of samples. Filtration was done in both forward and reverse direction. The data was first passed through high pass filter of order 2 and 8 Hz cut-off frequency and the resultant was passed through a low pass filter of order 2 and 30 Hz cut-off frequency.

### 2.4. Section IV: Feature Extraction

Power Spectral Density of each channel of the filtered signal was calculated. Each channel of filtered signal was divided into 4 windows of 62.5 ms each. A hamming window was created. The formula for hamming window is given in [22]

(2) $$\begin{array}{c} w\left(\;n\;\right)=0.54-0.46*\frac{\cos\left(2\pi n\right)}{N-1}, \end{array}$$

where *N* is the maximum number of points of the sampling window.

The window function was then multiplied with the signal to shape it into hamming window. Fast Fourier transform of the windowed signal was then calculated. Formula for Fast Fourier transform is given in [22]

(3) $$\begin{array}{c} X_{k}=\overset{N-1}{\underset{n=0}{\sum }}x_{n}e^{-i2\pi k\left(n/N\right)}\;k=0,\ldots ,N-1. \end{array}$$

The absolute value of the resultant is computed and divided by the normalization factor of the window.

The normalization factor of the window is given by [22]

(4) $$\begin{array}{c} U=\frac{1}{L}\overset{N-1}{\underset{n=0}{\sum }}{\left|\;w\left(\;n\;\right)\;\right|}^{2}. \end{array}$$

This gives us the Power Spectral Density of the window, which can be represented as in (5) [3]. After the PSD of each window is calculated, the corresponding values of all windows are added and averaged, leaving us with a vector of 8 constituents. Each value of this vector is then further divided by 2*π* to scale the values.

(5) $$\begin{array}{c} pxx=\frac{{\left|X\left(f\right)\right|}^{2}}{FsLU}, \end{array}$$

where Fs is sampling frequency, *L* is length of segment, *U* is window normalization constant given by (4), and *X*(*f*) is data after FFT.

**Figure 5:**
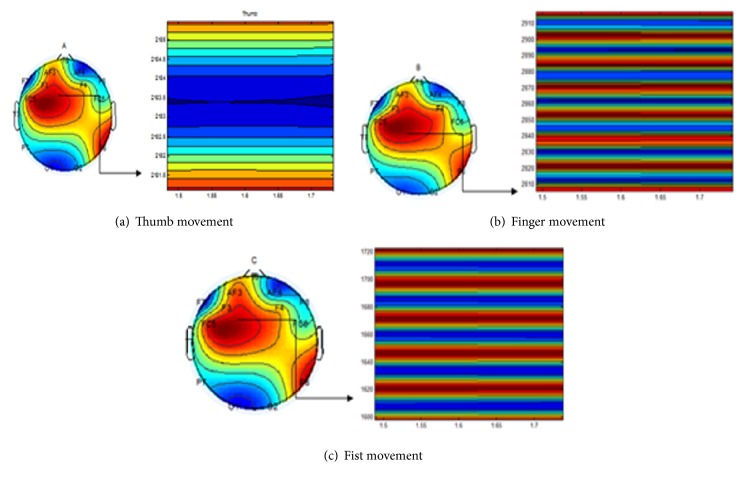
Topography plots of movements. FC5 and F3 electrodes have been magnified to show the difference in the topographies of the movements.

**Figure 6:**
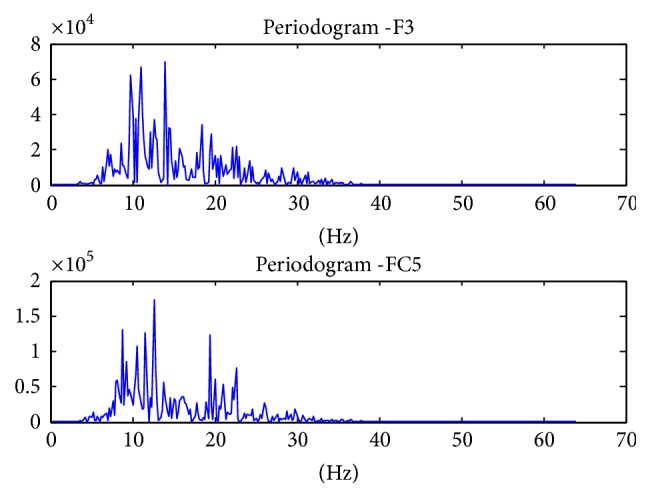
Finger movement periodogram of channels F3 and FC5.

**Figure 7:**
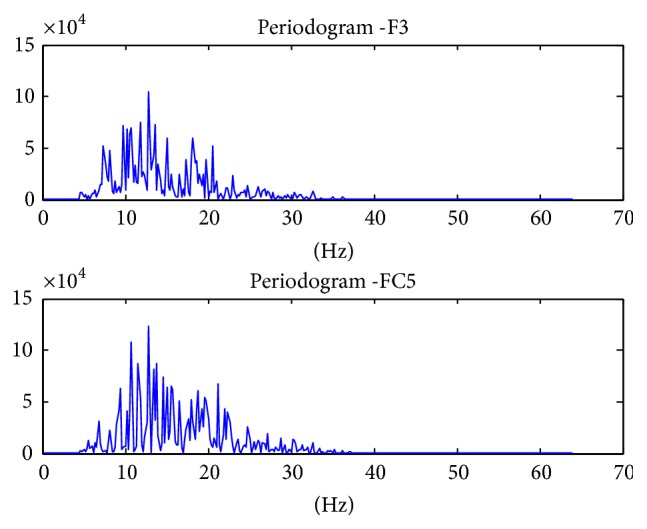
Thumb movement periodogram of channels F3 and FC5.

**Figure 8:**
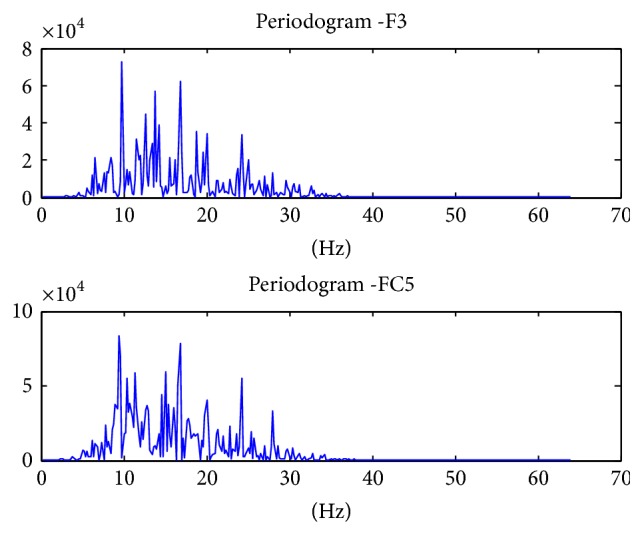
Fist movement periodogram of channels F3 and FC5.

The power values are averaged to give the band power of one channel of data. The process is repeated for all the 14 channels. In the end, we are left with a feature vector of 14 values, each representing the band power of 8–30 Hz frequency of the channel.  Figure 5 shows the topography plots of the raw data of randomly selected data samples of each movement. It can be seen that in each plot the electrodes F3 and FC5 contribute to rise in contours. These channels are basically located above the sensorimotor cortex. The contours due to these two electrodes have been magnified to show the difference in the topographies of the movements. The difference is also highlighted in the periodogram graphs that are shown from Figures 6–8 on channels F3 and FC5 of different movements. These graphs show the power concentration, in the 8–30 Hz band, of different movements.

### 2.5. Section V: Classification

As discussed earlier, our previous research had shown highest classification accuracy by using linear regression classifier. Therefore, for this research we used two-stage logistic regression classifier to calculate classification accuracy for three finger movements. For the logistic regression classifier, the probability of the first class is given by [23]

(6) $$\begin{array}{c} P\left(\;G=1\;\right)=\frac{\exp\left(B^{T}*F\right)}{\left(\exp\left(B^{T}*F\right)+1\right)}, \end{array}$$

where *F* is the feature vector and *B*^*T*^ are the coefficients of logistic regression.

The criterion for selection of class is [24, 25]

(7) Gx=class 1if  Pr>0.5Gx=class 1if  Pr<0.5.

**Figure 9:**
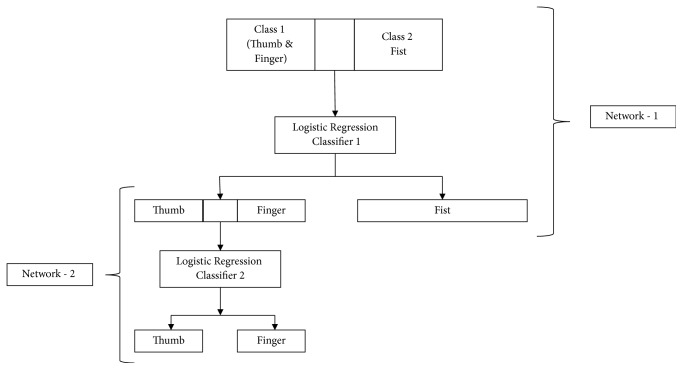
Two-stage logistic regression classifier used for the system.

In the two-stage model, the first classifier (referred to as network I) distinguished between class 1, which is thumb and finger movements, and class 2 which is fist movement. In the 2nd stage a second classifier (referred to as network II) distinguished between thumb and finger movement. The classifier model is shown in Figure 9. Training of the classifier was done using data set of all subjects (75% for training and 25% for testing) in “Weka” and coefficients of logistic regression were calculated for further use in classification using the embedded system. 31 randomly chosen samples for each movement were tested for classification in embedded system keeping in mind the data handling capability [26].

### 2.6. Section VI: Device Control

**Figure 10:**
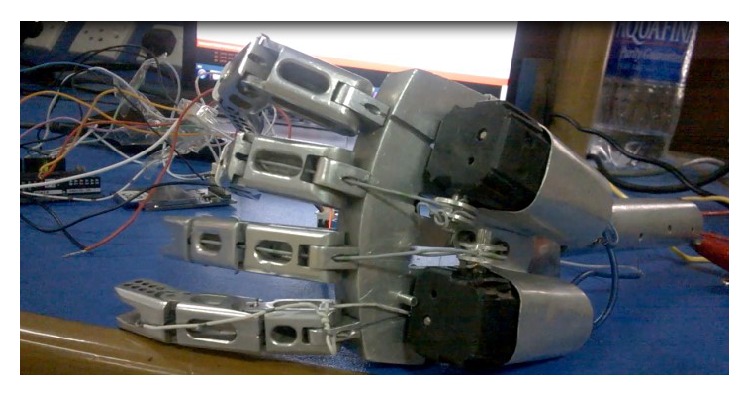
Prosthesis controlled by the embedded system.

Upper limb prosthesis used for this research was developed in the department for carrying out the experiments.  Figure 10 shows a picture of upper limb prosthesis.

Prosthesis contains two motors connected to two fingers and placed at the palm. Finger joints are connected with each other with the help of a flexible metal wire which is connected with a motor. Motor rotation will cause winding or unwinding of the flexible metal wire resulting in opening or closing of fingers. Both motors were connected to motor drive which was taking command signal from Arduino Uno. The embedded system generated a control signal based on the classification of finger movements. This control signal was sent to the motor drive circuitry to actuate the motor for desired motion. One of the motors is attached to Output Pins 4 and 6 (for thumb movement) and the other is attached to Output Pins 2 and 3 (for finger movement). Motion of motors according to classification is shown in Table 6.

## 3. Results

To train the two-stage logistic regression classifier data set of all subjects (category I and category II) is used as discussed in Section 2.5. 75% data is used for training and 25% data is used for testing. “Weka” (data mining software) was used and coefficients of logistic regression were calculated for further use in embedded system.

Results of our research comprise two categories. In category I (subject having a habit of high involuntary eye blinking frequency), 31 randomly chosen data samples from each movement were used for testing using embedded system. In category II (subjects other than category I), 31 randomly chosen data samples from each movement were used for testing using embedded system.

Table 7 shows the classification accuracy of each stage of classifier (network I and network II) using data set of all subjects (category I and category II) as discussed in Section 2.5.

Table 8 shows the confusion matrix of category I data set tested over 31 randomly chosen samples for each movement and Table 9 shows the category II data set tested over 31 randomly chosen samples for each movement.

Table 10 shows the per class classification accuracy of randomly chosen samples from category I and category II. Percentage accuracies are calculated on the basis of confusion matrices shown in Tables 8 and 9.

## 4. Discussion

The aim of this research was to investigate the design of an embedded system for control of upper limb prosthesis as an extension of our previous research. As evident from Table 1, research using BCI system for control of prosthesis is focused on spatially distant motor movements. Our focus in this research was to control prosthesis with finger movements which have less spatial distance as compared to earlier researches.

Finger movements have the same origin in brain leading to extremely small spatial difference between them. Our endeavor was to pick up the small difference of brain activity recorded in the form of electrical potential and classify it with higher accuracy. It was seen from the topography plots shown in Figure 5 that finger movements have the same origin in brain. The minor differences in the topographies were highlighted when the data under electrodes F3 and FC5 was interpolated and plotted in a magnified manner.

Band power of Power Spectral Density of Mu and Beta Rhythms was chosen as feature vectors. PSD is used as a feature vector in 70% of the research which focuses on motor controls. The periodogram of the movements was plotted for the channels above sensorimotor cortex to visualize the differences.

The development of the embedded system was focused on to design a control for upper limb prosthesis that has small size and light weight and is easy to carry onboard system for prosthesis user. The acquired data was saved on SD card rather than on the controller to depict a real time data processing and classification. 70% mean classification accuracy was achieved with 2-stage logistic regression classifier using an Arduino Uno based embedded system. It should also be noted that the index and middle finger combined movement could not be classified with higher accuracies since the spatial distance is very less. This accuracy needs to be increased further for developing better control of prosthesis and practical implementation.

The challenge is to create an online model that can process and classify the real time data. However, implementation of the model on patients requires a more robust system suggested subsequently. A headset with greater number of channels especially above the motor cortex is required for recording more comprehensive signals. It is also recommended to use an embedded system with higher computational speed which can process signals in real time.

It can also be seen from our results that eye blinking during data acquisition induces ocular artefacts which result in lower classification accuracies. For a better and higher classification accuracies a signal with higher signal to noise ratio is required. Different techniques for removing ocular artefact may be used for future work.

Overall the research shows that upper limb prosthesis control can be achieved even with signals that are taken from closely situated body part with an average accuracy of 70% (calculated on the basis of classification accuracy of category II randomly chosen samples as mentioned in Table 10).

## 5. Conclusion

The designed embedded system in this research is capable of controlling the prosthesis based on the model developed earlier. A two-stage classifier has been designed and implemented over the embedded systems. The classifier is capable of distinguishing between three movements of finger, thumb, and fist. The mean classification accuracy of 70% is attained by the developed system. Further work to improve the classification accuracy using advanced embedded system can be undertaken for enhanced prosthesis control.

## Figures and Tables

**Table 1:** Examples of research for prosthesis or cursor control using motor imagery.

Index	Year	Research	Protocol	Accuracy	Device Control
1	2011	“Real-time control of a prosthetic hand using human electrocorticography signals” [4]	ECoG of three movements of left hand (grasping motion, hand opening motion, scissor type motion)	69.2%	Prosthesis control

2	2008	“Control of an electrical prosthesis with an SSVEP-based BCI” [5]	Steady-state visual evoked potentials	Between 44% and 88% of four patients	Control of two-axes electrical hand prosthesis

3	2012	“Target Selection with Hybrid Feature for BCI-Based 2-D Cursor Control” [6]	Hybrid feature from motor imagery and the P300 potential. Target selection by focusing and direction control by left-right hand motor imagery.	93.99%	Online control of cursor on a monitor screen

4	2013	“Quadcopter control in three-dimensional space using a noninvasive motor imagery-based brain–computer interface” [7]	Motor Imagery of left or right hand movement for 1D cursor movement left and right. For 2D movement move cursor up by imagining squeezing or curling both hands and to move the cursor down through the use of a volitional rest.	Between 69.1% and 90.5% for 5 subjects	Quadcopter control

5	2014	“Simultaneous Neural Control of Simple Reaching and Grasping with the Modular Prosthetic Limb Using Intracranial EEG” [8]	Intracranial electroencephalographic (iEEG) signals of subject who made reaching and grasping movements to identify task-selective electrodes	Independently executed overt reach and grasp movements for (Subject 1, Subject 2) were (0.85, 0.81) and (0.80, 0.96), respectively, during simultaneous execution they were (0.83, 0.88) and (0.58, 0.88), respectively	Dexterous robotic prosthetic arm

6	2009	“Decoding human motor activity from EEG single trials for a discrete two-dimensional cursor control” [9]	Four motor tasks (sustain or cease to move right or left hand)	Average accuracy of 85.5 ± 4.65% with physical motor movement	2D cursor movement

**Table 2:** Attributes of embedded system.

	Attribute	Specification
(1)	Memory	32 kB

(2)	Debug ability	(i) In-System Programming by On-chip Boot Program (ii) True Read-While-Write Operation

(3)	Reliability	Data Retention: 20 years at 85°C/100 years at 25°C

(4)	Throughput	Up to 20 MIPS Throughput at 20 MHz

(5)	Testability	(i) In-System Programming by On-chip Boot Program (ii) True Read-While-Write Operation

(6)	Response	Speed Grade: (i) 0–4 MHz @ 1.8–5.5 V (ii) 0–10MHz @ 2.7–5.5 V (iii) 0–20 MHz @ 4.5–5.5 V

**Table 3:** Connection between SD card and Arduino.

SD card (Pin)	Arduino Uno (Pin)
5 V	5 V
Ground	Ground
CS	Pin 10
MOSI	Pin 11
MISO	Pin 12
SCK	Pin 13

**Table 4:** High pass filter coefficients.

Vector	Index 1	Index 2	Index 3
**a**	1	−1.4542	0.5741
**b**	0.7571	−1.5142	0.7571

**Table 5:** Low pass filter coefficients.

Vector	Index 1	Index 2	Index 3
**a**	1	−0.1151	0.1739
**b**	0.2647	0.5294	0.2647

**Table 6:** Motion of motors according to classification.

Classification of finger movement	State of motor attached to Output Pins 4 and 6	State of motor attached to Output Pins 2 and 3
Thumb movement	On	Off
Finger movement	Off	On
Fist movement	On	On

**Table 7:** Network classification accuracy of a two-stage logistic classifier network.

Network number	Classification accuracy
Network 1 (Class 1-Thumb + Index Finger and Class 2- Fist)	74%
Network 2 (Class 1-Thumb and Class 2- Index Finger)	76%

**Table 8:** Confusion matrix of category I data set.

Class	Class predicted by 2-stage logistic regression classifier		
	Thumb	Index Finger	Fist
Thumb	13	12	6
Index Finger	8	16	7
Fist	8	9	14

**Table 9:** Confusion matrix of category II data set.

Class	Class predicted by 2-stage logistic regression classifier		
	Thumb	Index finger	Fist
Thumb	20	9	2
Index finger	4	24	3
Fist	5	5	21

**Table 10:** Per class accuracy.

Movement class	Classification accuracy of category I	Classification accuracy of category II
Thumb	42%	65%
Index finger	51%	77%
Fist	45%	68%
